# An outbreak of acute respiratory infection at a training base in Beijing, China due to human adenovirus type B55

**DOI:** 10.1186/s12879-020-05258-2

**Published:** 2020-07-23

**Authors:** Guilan Lu, Xiaomin Peng, Renqing Li, Yimeng Liu, Zhanguo Wu, Xifeng Wang, Daitao Zhang, Jiachen Zhao, Ying Sun, Li Zhang, Peng Yang, Quanyi Wang

**Affiliations:** 1 Institute for Infectious Disease and Endemic Disease Control, Beijing Center for Disease Prevention and Control, Beijing Research Center for Preventive Medicine, 16# He Ping Li Middle Street, Dongcheng District, Beijing, 100013 China; 2 Daxing District Center for Disease Prevention and Control, Beijing, 102600 China

**Keywords:** Outbreak, Human adenovirus, Acute respiratory infection, Phylogenetic analysis, Whole genome sequencing

## Abstract

**Background:**

Twelve students experienced symptoms of acute respiratory infection (ARI) at a training base in Beijing from August 26 to August 30, 2015. We investigated the cause of this ARI outbreak.

**Methods:**

In partnership with the local center for disease control, we collected a total of twelve pharyngeal swab specimens as well as demographic information for the affected patients. We used multiplex real-time PCR to screen for sixteen common respiratory viruses in these samples. To isolate HAdV, we inoculated Hep-2 cells with the human adenovirus (HAdV)-positive samples and then carried out sequencing and phylogenetic analysis of the hexon, fiber, and penton genes of the isolated adenoviruses. In addition, we analyzed the entire genome of one strain isolated from the index case to identify single-nucleotide substitutions.

**Results:**

We identified ten HAdV-positive students using multiplex real-time PCR. None of the students were co-infected with other viruses. We successfully isolated seven HAdV strains from the pharyngeal swab specimens. The coding sequences of the hexon, fiber, and penton genes of these seven HAdV strains were identical, suggesting that they represented seven strains from a single virus clone. One HAdV isolate obtained from the index case, BJDX-01-2015, was selected for whole genome analysis. From this isolate, we obtained a 34,774-nucleotide sequence. The genome of BJDX-01-2015 clustered with HAdV-B55 in phylogenetic analyses and had 99.97% identity with human adenovirus 55 isolate HAdV-B/CHN/BJ01/2011/55 (GenBank accession no. JX491639).

**Conclusions:**

We identified HAdV-B55 as the strain associated with the August 2015 ARI outbreak at a training base in Beijing. This was the first reported outbreak in Beijing due to HAdV-B55. Continuous surveillance of respiratory adenoviruses is urgently needed to understand the epidemiological and evolutionary features of HAdV-B55, and an epidemiological modeling approach may provide further insights into this emerging public health threat. Furthermore, the clinical laboratory data from this outbreak provides important reference for the clinical diagnosis and may ultimately aid in informing the development of strategies to control and prevent respiratory tract infections caused by HAdV-B55.

## Background

Human adenoviruses (HAdVs) cause a wide variety of clinical manifestations, including respiratory tract infection, gastroenteritis, kerato-conjunctivitis, acute hemorrhagic cystitis, nephritis, hepatitis, and encephalitis [1–3]. HAdVs are responsible for 2–5% of all respiratory illnesses and for 4–10% of pneumonias in children [4, 5]. Most HAdV infections are mild, self-limiting, and indistinguishable from other viral infections. However, the illnesses caused by HAdVs can be severe or even fatal and can result in substantial morbidity [6, 7]. Outbreaks of HAdV-associated acute respiratory infection (ARI) usually occur in healthy children or in adults in enclosed or crowded settings [1, 8]_._

HAdV was first reported as a viral pathogen in 1953 [6]. Since this initial identification, HAdVs have been classified into seven species (A to G), and the Human Adenovirus Working Group has identified 90 HAdV types as of July 2018 (http://hadvwg.gmu.edu/) [7–12]. Adenoviruses are non-enveloped icosahedral particles that contain linear double-stranded DNA genomes with sizes ranging from 26 to 45 kb. Adenovirus genomes are characterized by inverted terminal repeat sequences (ITRs) with sizes ranging from 36 to over 200 bp [12]. The HAdV viral capsid, which surrounds the genome, is composed of three major proteins: hexon, penton base, and fiber [13].

Different HAdV species display various tissue tropisms that correlate with the different clinical symptoms of infection [14]. HAdV species A has often been associated with the gastrointestinal tract, whereas species B (HAdV-3, 7, 14, and 55), C (HAdV-1, 2, 5, and 6), and E (HAdV-4) are known to cause respiratory tract infections. Species D (HAdV-8, 19, and 37) commonly causes adenoviral kerato-conjunctivitis. Species F variants, including HAdV-F40 and-F41, and species G variant HAdV-G52 are mainly associated with gastrointestinal tract infections [2].

HAdVs have been associated with previous outbreaks of ARI. HAdV-B3 and HAdV-B7 cause frequent outbreaks in the United States [15]. In Asia, the prevalence of HAdVs in patients with ARIs has ranged from 0.8 to 11.30% [16–20]. Recently, Guo et al. (2012) reported that HAdV-B3, HAdV -B7, HAdV -B11, and HAdV -B14 were the most frequently detected virus strains among patients with acute adult adenovirus infections in Beijing from May 2005 to July 2010 [16].

In China, HAdV-B3 and HAdV-B7, two HAdV species B subtypes, are common causes of epidemic ARI outbreaks [21–24]. In 2006, an ARI outbreak occurred in Qishan, Shaanxi Province, China. A re-emergent isolate of HAdV-B55 (QS-DLL), originally described as HAdV-11a and fully characterized in 2009, was reported to be the cause of this outbreak [25]. This re-emergent HAdV-B55 was shown to have evolved from a hexon recombination between HAdV-B11 and HAdV-B14 [9, 25]. Since its characterization, HAdV-B55 has been associated with several respiratory infection outbreaks and is known to be responsible for severe respiratory diseases [9, 25–28].

Here we describe an outbreak of ARI caused by HAdV-B55 at a training base in the Daxing District of Beijing, China. To help identify the causative pathogen, we collected pharyngeal swab specimens from the affected students and carried out molecular detection and typing, phylogenic analysis, and whole-genome sequencing. This is the first reported outbreak of ARI in Beijing due to HAdV-B55.

## Methods

### The training base where the HAdV-B55 outbreak took place

On August 31, 2015, local public health authorities were informed about an outbreak of ARI among young students at a training base located in the Daxing District of Beijing. The training base consisted of two three-floor buildings for teaching and three three-floor dormitories with eight persons to a room. The training base recruits only male middle school graduates. Approximately 3000 students majoring in Mathematics, Chinese, and English were enrolled in a total of sixty classes. The training base employs 100 full-time staff members.

### Epidemiological investigation

On August 26, 2015, one student reported symptoms of an ARI and had a body temperature of 38.4 °C. By August 30, a total of 12 ARI patients from the same class were reported by the local hospital. For the purposes of our analysis, we defined ARI cases as individuals with a body temperature over 38.0 °C and with at least one symptom of a respiratory tract infection, such as cough or sore throat. On August 31, the Daxing District Center for Disease Prevention and Control (CDC) began an epidemiological investigation, collecting demographic, clinical and laboratory data. Under the guidance of the CDC, the training base took precautionary measures, including quarantining the affected students, carrying out a routine cleaning and disinfection of living quarters, and morning body temperature checks. No further new cases were reported by September 08, 2015.

### Patients and samples

Pharyngeal swab samples were obtained from each of the twelve students infected in this ARI outbreak. The specimens were collected in 3ml vials containing viral transport medium and quickly transported on ice to the laboratory of the Daxing District CDC. The specimens were stored at − 80 °C until further use. Patient information and laboratory results are shown in Table 1.

**Table 1:** Patient information and laboratory results

ID No.	Onset date (m/d/y)	Specimen Collection date (m/d/y)	Clinical symptoms									
			Body temperature (°C)	Cough	Sore throat	Body aches	Headache	Nasal congestion	Rhinorrhea	Conjunctival hyperemia	Diarrhea	White blood cell count (10^9^/L)
1	08/26/2015	08/31/2015	38.4	–	+	+	+	–	–	–	–	7.8
2	08/26/2015	08/31/2015	39.0	–	–	+	+	–	–	–	–	5.2
3	08/27/2015	08/31/2015	38.5	+	–	–	+	+	–	–	–	9.1
4	08/28/2015	08/31/2015	39.5	–	+	–	+	–	–	–	–	5.7
5	08/28/2015	08/31/2015	39.6	–	+	+	+	+	–	–	–	7.0
6	08/29/2015	08/31/2015	40.0	+	+	–	+	–	–	–	+	5.0
7	08/29/2015	08/31/2015	38.8	–	+	–	–	–	–	–	–	6.4
8	08/30/2015	08/31/2015	38.2	–	+	–	+	–	–	–	–	8.3
9	08/30/2015	08/31/2015	39.4	–	–	+	–	–	–	–	–	9.3
10	08/30/2015	08/31/2015	39.0	–	+	–	–	–	–	–	–	/
11	08/30/2015	08/31/2015	38.6	–	+	+	+	–	–	–	–	7.9
12	08/29/2015	08/31/2015	39.6	–	+	–	–	–	–	–	–	/

*ID No*. Identification number; +, yes; −, no; /, not tested

### Respiratory virus detection

Nucleic acids were extracted from 140 μl of each of the clinical samples using QIAamp Viral RNA mini Kits (Qiagen, Hilden, Germany) according to the manufacturer’s instructions. Pharyngeal swab specimens were screened for 16 common respiratory pathogens via real-time PCR multiplex assays using commercial kits (Uninovo Biological Technology, Zhenjiang, China) as described by Shi W., et al. [29]. The 16 pathogens assayed using this approach were influenza virus A (H3), pandemic influenza virus A (H1N1), influenza viruses A and B (Flu A and B), parainfluenza viruses 1, 2, 3, and 4 (PIV1, 2, 3, and 4), human metapneumovirus (hMPV), human bocavirus (HBoV), human coronavirus OC43/NL63, 229E/ HKU1, human respiratory syncytial virus (HRSV), human rhinovirus (HRhV), and HAdV.

### HAdV isolation and typing

Hep-2 cells were inoculated with HAdV PCR-positive specimens and cultured in high-glucose Dulbecco’s Modified Eagle Medium (Gibco, NY, USA) containing 2% fetal bovine serum (Gibco, NY, USA), 100 U/mL penicillin (Gibco, NY, USA), and 100 mg/mL streptomycin (Gibco, NY, USA) at 37 °C in a 5% CO_2_ incubator for 1 week following standard protocols [25]. The cultured cells were checked regularly for cytopathic effects (CPE) and harvested when cytopathic effects (CPE) were observed. Cultures with CPE were screened for specific HAdVs as described by Kim C et al. [30].

Molecular typing of HAdVs was performed via conventional PCR using specific primers targeting the complete coding sequences of the hexon, fiber, and penton genes [31]. Viral DNA was extracted from cultured medium using QIAamp RNA mini kits (Qiagen, Hilden, Germany) according to the manufacturer’s instructions [32]. Conventional PCR was conducted using high-fidelity DNA polymerase (Takara, Dalian, China) according to the manufacturer’s instructions. The hexon, fiber, and penton genes of HAdV were amplified as described previously [31]. For the hexon and penton genes, the PCR protocol was: 94 °C for 5 min., followed by 35 cycles of 50 s. at 94 °C, 50 s. at 55 °C, 3 min. at 72 °C, and a final extension step of 72 °C for 10 min. The PCR protocol for amplification of fiber gene fragments was identical, except for the annealing temperature, which was 52 °C instead of 55 °C. The amplified PCR products were excised from agarose gels, purified using an Axyprep DNA gel extraction kit (Axygen, Hangzhou, China), and bi-directionally sequenced using the Sanger sequencing method by Invitrogen Biotechnology Co., Ltd. (Invitrogen, Beijing, China) with an ABI 3730 DNA Analyzer (Applied Biosystems, Austin, TX, USA).

### Whole-genome sequencing

To further analyze mutations in the genome sequences of the viruses isolated in this ARI outbreak, we sequenced the whole genome of one isolate from the index case using the Sanger method. Targeted 1-2 kb segments that covered the entire genome with overlapping sequences of about 200 bp were amplified by PCR. The 5′ and 3′ ITRs of the genome were amplified and cloned into a plasmid T-vector and then sequenced. A set of 47 pairs of primers was designed in-house to amplify the whole genome according to the reference sequence (GenBank accession no. FJ643676) and then used for separate PCRs. Primer sequences are available upon request.

Whole-genome sequencing segments were amplified using high-fidelity polymerase (Takara, Dalian, China) using 1.0 mM of each primer. PCRs were carried out using a BioRad thermocycler (Applied Biosystems, Austin, TX, USA) with the following protocol: 94 °C for 5 min., followed by 35 cycles of 50 s. at 94 °C, 50 s. at appropriate annealing temperature for separate primers, 3 min. at 72 °C, and a final extension step of 72 °C for 10 min.

The amplified segments were purified and bi-directionally sequenced. Gaps and ambiguous sequences were PCR-amplified using new primers and re-sequenced. DNA sequence fragments were assembled using the SeqMan program implemented in DNASTAR Lasergene 7.0 (DNASTAR, Inc. Madison, WI) into a single contig. The genomic sequence determined in this study was deposited in GenBank under accession number MK886831.

### Sequence alignment and phylogenetic analysis

Nucleotide sequence homologies were identified using the Basic Local Alignment Search Tool (BLAST, https://blast.ncbi.nlm.nih.gov/). Multiple nucleotide sequence alignments were performed using the ClustalW program implemented in BioEdit. Comparisons between the whole genome sequence of the BJDX-01-2015 virus strain and those of other types of HAdVs were generated using CLC Genomics Workbench (Qiagen, Hilden, Germany).

Phylogenetic trees were constructed using the maximum likelihood method in the MEGA program (Version 5.05). One thousand bootstrap replications were used to estimate distances. Bootstrap values greater than 70% are shown for selected nodes in Fig. 2 (a-d). Whole-genome sequences and hexon, fiber, and penton gene sequences from other HAdVs were downloaded from GenBank on April 3, 2019.

## Results

### Outbreak description

**Fig. 1:**
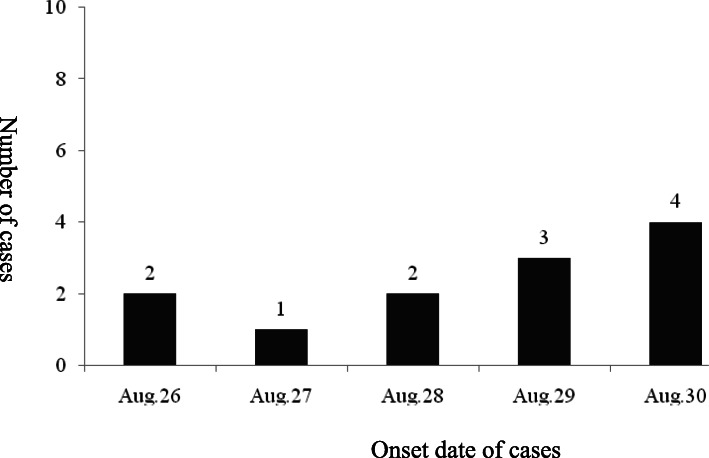
Case distribution during a 2015 outbreak of HAdV-B55 in Beijing. Left *Y*-axis, number of cases; right *Y*-axis, onset date of cases

On August 26, 2015, a 15-year old male student developed a case of ARI, with a peak body temperature of 38.4 °C. By August 31, a total of 12 male students were infected. No females were infected. The mean age of the infected students was 15.4 years (median, 15 years; range, 14–17 years). The distribution of daily cases is shown in Fig. 1**.** Reported students were from the same class but living in six different dormitory rooms. The outbreak lasted for 6 days.

### Clinical symptoms of the infected students

The clinical symptoms of the infected students are described in Table 1**.** All 12 infected individuals had a fever; 9 (75%) students had a sore throat, and 8(66.67%) students had a headache. Other symptoms reported by the patients were cough, body ache, stuffy nose, and diarrhea. All affected students had normal white blood counts. Most of the infected students were treated in outpatient clinics; only one patient, who had non-severe pneumonia, was hospitalized.

The index case (ID No.1) was diagnosed as having an upper respiratory infection with a peak temperature of 38.4 °C, a neutrophil count of 72.2% (normal range, 50–70%), percentage of lymphocytes of 22.2% (normal range, 20–40%), and white blood cell count of 7.8 × 10^9^/L (normal range, 4.0–10.0 × 10^9^/L).

The hospitalized student (ID No.6) had a peak temperature of 40.0 °C accompanied by a cough, headache, sore throat, and diarrhea. He had a neutrophil count of 78.6% (normal range, 50–70%), a percentage of lymphocytes of 17.8% (normal range, 20–40%), and a white blood cell count of 5.04 × 10^9^/L (normal range, 4.0–10.0 × 10^9^/L). A chest x-ray showed patchy shadows on the right lung of the hospitalized student.

### Multiplex-PCR detection

A total of 12 respiratory samples were obtained from the 12 students. Ten specimens were shown to be HAdV-positive using multiplex PCR (Uninovo Biological Technology, Jiangshu, China). None of the 10 HAdV-positive patients were co-infected with other respiratory viruses.

### Virus isolation and HAdV typing

To isolate viruses, we inoculated Hep-2 cells with the HAdV-positive samples and isolated seven HAdV virus strains. Using typing primers, which allow for the determination of viral type, we sequenced the hexon, fiber, and penton genes in the seven HAdV strains [31].

The hexon (2841 bp), fiber (978 bp), and penton (1674 bp) sequences from the seven HAdV isolates were 100% identical, suggesting that this outbreak was caused by a single viral strain. We then compared the hexon, fiber, and penton sequences of one of the viral strains isolated from the index case (ID No.1; referred to as BJDX-01-2015), with other HAdV-B55, HAdV-B11 and HAdV-B14 strains. Based on BLAST analysis, the hexon, fiber, and penton genes were 99.6, 100, and 100% identical to the genes of the HAdV-B55 reference strain QS-DLL in China (GenBank accession number FJ643676), respectively,. The hexon, fiber, and penton genes were each 100%, identical to the genes of the isolate HAdV-B/CHN/BJ01/2011/55 (GenBank accession no. JX491639; Table 2), which was identified from a single patient in Beijing with severe community-acquired pneumonia (CAP) who was infected with HAdV-B55. One of the seven strains, BJDX-01-2015, was selected for further whole-genome studies.

**Table 2:** Nucleotide identities of BJDX-01-2015 compared with other HAdV-B55, HAdV-B11, and HAdV-B14 strains based on the hexon, fiber and penton genes

Type	Strain name	GenBank accession number	Hexon	Fiber	Penton
HAdV55	HAdV-B/CHN/BJ01/2011/55	JX491639	100%	100%	100%
	QS_DLL	FJ643676	99.96%	100%	100%
	CQ-1657 /2011	JX123028	99.96%	100%	100%
	Shanxi/QZ01/ 2011	KJ883522	99.96%	99.90%	100%
	Hebei/BD6729/2013	KJ883521	99.96%	99.80%	99.94%
	SGN1222	FJ597732	99.93%	99.90%	100%
	Yunnan/ KM04 /2016	KY002685	99.89%	99.90%	100%
	South Dakota/6380/1997	FJ841899	99.93%		
	Spain/273/1969	FJ841900	99.58%		
	South Dakota/6380/1997	FJ841907		100%	
	Spain/273/1969	FJ841908		99.90%	
HAdV11	Slobitski	AF532578	98.07%	94.39%	97.69%
HAdV14	de Wit	AY803294	92.67%	99.59%	99.64%
	CHN 2012	JX892927	92.57%	99.08%	99.64%

### Whole-genome sequencing

We obtained and assembled the full genome of the index case isolate, BJDX-01-2015. The sequence of this genome has been deposited in GenBank (accession number MK886831). The complete genome of BJDX-01-2015 was 34,774 nucleotides in length and had 137-bp inverted terminal repeat sequences in the 5′- and 3′- untranslated regions (5′-UTR and 3′-UTR).

### Phylogenetic analysis of the HAdV-B55 strain

**Fig. 2:**
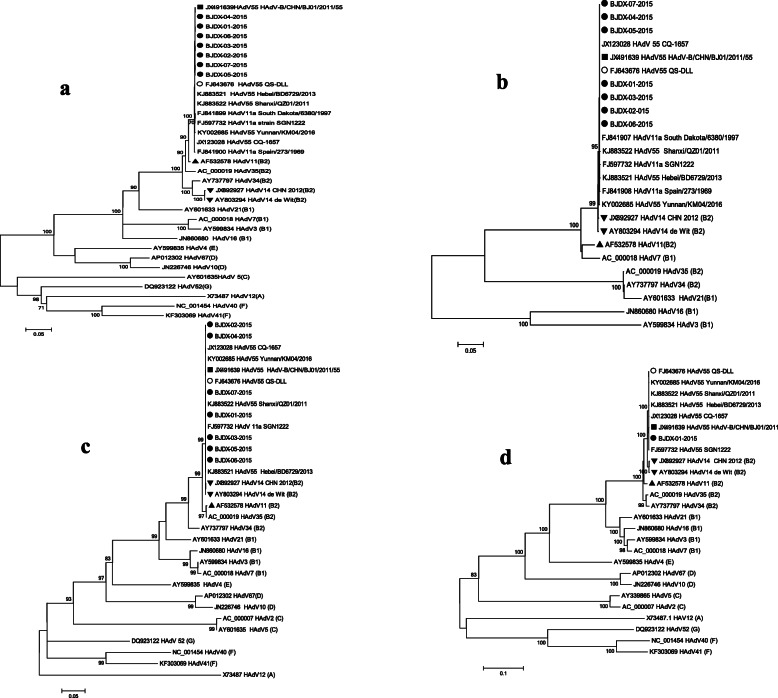
Based on the complete sequences of (**a**) hexon, (**b**) fiber, (**c**) penton and (**d**) complete genome, the maximum likelihood trees were constructed using the Tamura-Nei model (Gamma distributed with Invariant sites (G + I)), the Hasegawa-Kishino-Yano model (Gamma distributed (G)), the Tamura-Nei model (Gamma distributed with Invariant sites (G + I)), the Tamura-Nei model (Gamma distributed with Invariant sites (G + I)), respecitely, with 1000 bootstrap replicates using MEGA 5.0. Bootstrap-supported values greater than 70% are indicated at each node. The scale bar indicates the number of nucleotide substitutions per site. The species of adenovirus is specified in parentheses at the end of the strain information. ●, isolate from this outbreak; ■, HAdV-B/CHN/BJ01/2011/55; ○,QS-DLL strain;▲, HAdV-B11 isolates;▼, HAdV-B14 isolates

To investigate the genetic relationships between isolate BJDX-01-2015 and other HAdV strains, we constructed phylogenetic trees (Fig. 2 (a-d)) using the maximum likelihood method based on the complete hexon, fiber, and penton gene sequences of strain BJDX-01-2015 and other HAdV strains. All phylogenetic trees showed that BJDX-01-2015, associated with this outbreak, clustered with HAdV-B55. The BJDX-01-2015 hexon gene also clustered with HAdV-B11 (GenBank accession number AF532578), while the fiber and penton genes and the full BJDX-01-2015 genome clustered with HAdV-B14 (GenBank accession number AY803294 and JX892927).

### Whole-genome alignment and mutation analysis

**Fig. 3:**
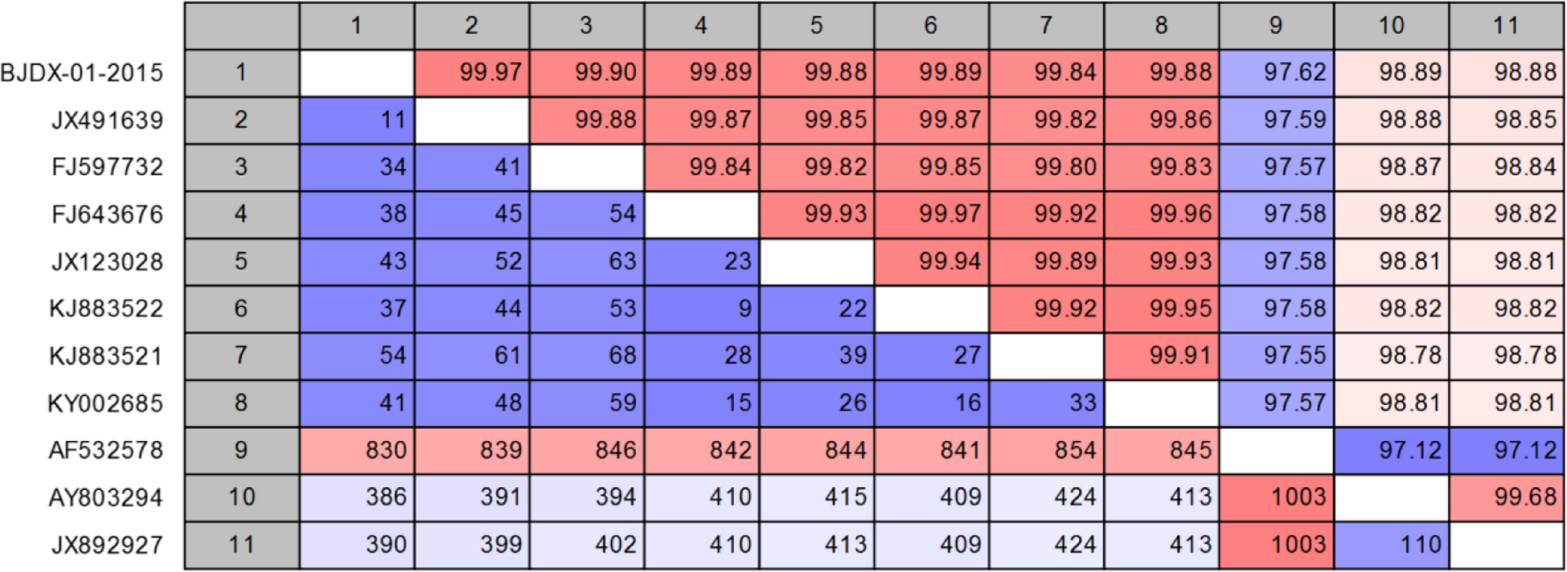
Percent nucleotide identity and differences in the genome of BJDX-01-2015 relative to strains of HAdV-B55, HAdV-B11, and HAdV-B14. The numbers above the white grids represent the percent nucleotide identity, while those below the grids represent the nucleotide difference values calculated by CLC Genomic Workbench

Comparisons of the whole genome sequence of the BJDX-01-2015 virus strain with other HAdV-B55, HAdV-B11 and HAdV-B14 strains are shown in Fig. 3. The genome of BJDX-01-2015 was 99.97% identical to that of the HAdV-B/CHN/BJ01/2011/55 (GenBank accession number JX491639) strain, having 11 different nucleotides, and 99.89% identical to the strain QS-DLL (GenBank accession number FJ643676), having 38 different nucleotides. BJDX-01-2015 shared 97.62% of its genome with the HAdV-B11 reference strain (GenBank accession number AF532578), and 98.89 and 98.88% of its genome with two HAdV-B14 strains (GenBank accession numbers AY803294 and JX892927; Fig. 3).

**Fig. 4:**
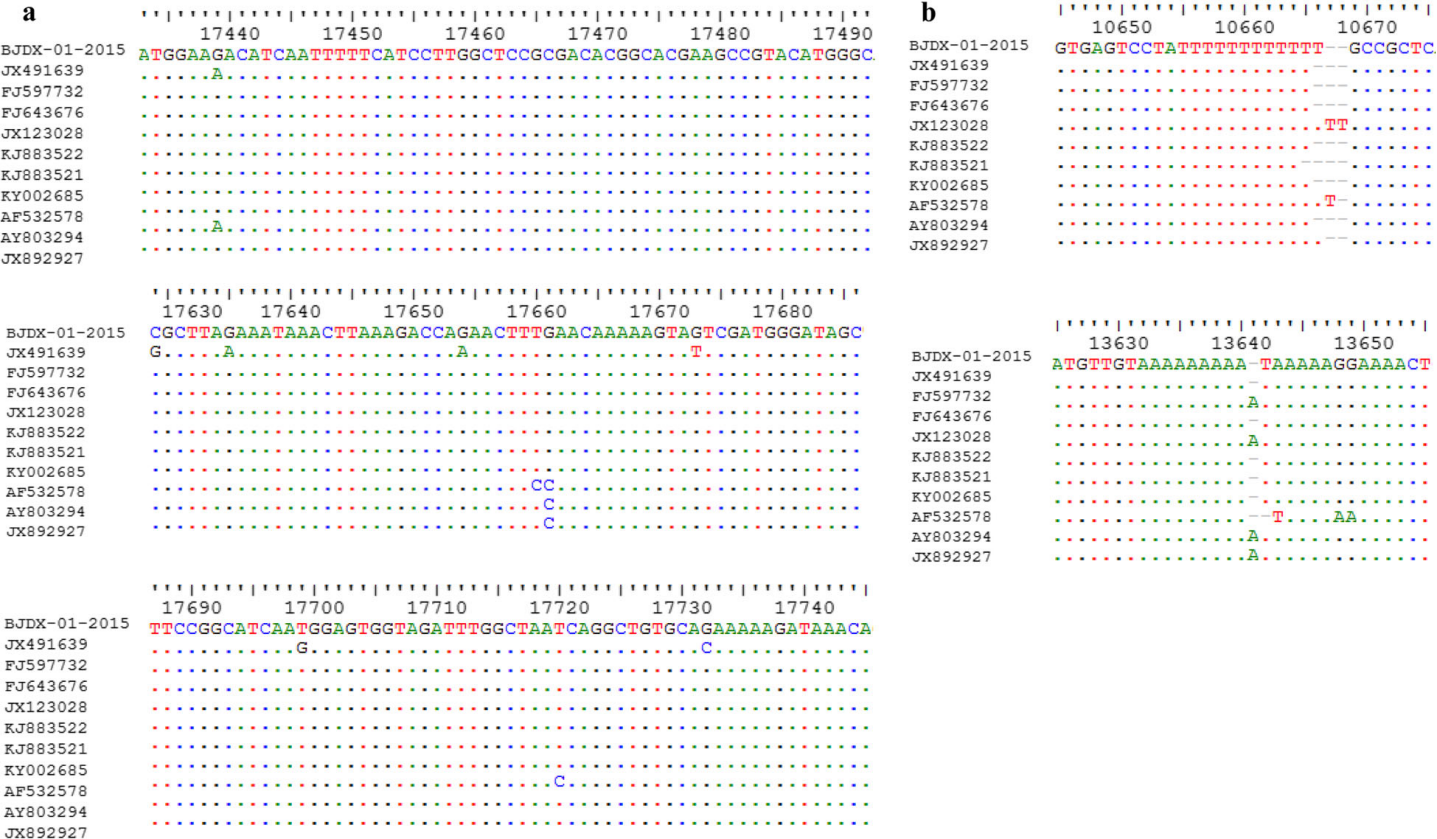
(**a**) Alignment analysis on the coding sequence of protein VI among HAdVs. ‘.’ represents identical bases in the sequence alignment. (**b**) Different numbers of poly “T” and poly “A” tracts were observed in the aligned genomes of the HAdV-B55, HAdV-B11, and HAdV-B14 strains. ‘.’ and ‘-’ represent identical bases and deletions in the sequence alignment, respectively

We then generated alignments between the genome sequence of strain BJDX-01-2015 and those of strains HAdV-B55, HAdV-B11, and HAdV-B14. Most of the nucleotide differences in BJDX-01-2015 relative to HAdV-B/CHN/BJ01/2011/55 (GenBank accession number JX491639) were observed in the coding region of protein VI (Fig. 4a), where we found seven nucleotide changes and six amino acid substitutions. We also found different numbers of poly “T” and ploy “A” tracts (Fig. 4b).

## Discussion

This study describes an ARI outbreak with 12 students infected at a training base in the Daxing District of Beijing, China, in August 2015. Based on epidemiological and laboratory investigation, we confirmed that the etiologic pathogen of this ARI outbreak was HAdV-B55.

HAdV-B55 has received additional attention in recent years. HAdV-B55 is a recombinant virus, and it is associated with more severe acute respiratory diseases than other types of HAdVs [33]. HAdV-B55 was originally described as HAdV-B11a based on earlier putative, sporadic occurrences in Spain (1969) [34], Turkey (2004) [35], and Singapore (2005) [36]. HAdV-B55 was also identified as a re-emergent acute respiratory disease pathogen after a recent outbreak in Qishan County, Shaanxi Province, China in 2006 [9, 25]. Subsequent analysis revealed that HAdV-B55 consists of an HAdV-B14 backbone and a partial HAdV-B11 hexon gene. This re-emergent virus exhibited a neutralizing antigen epitope of HAdV-B11 and the pathogenic properties of HAdV-14. Therefore, this virus was renamed HAdV-B55 [9, 37].

In China, an increasing number of outbreaks of HAdV-B55 have been reported since March 2006 [25]. Epidemic outbreaks of HAdV-B55 have occurred in military camps, schools, and even hospitals in Hebei Province (February 2012) [38], Tianjin City (January 2013) [27], Beijing (2015; this study), Guangzhou City (2016) [28], Tibet, Sichuan and Yunnan Provinces (2016) [39].

In the 2006 HAdV-B55 outbreak in China, a total of 254 patients were infected, and one died [25]. Although the virus spread quickly in the 2015 outbreak reported here, only 12 individuals were infected, including one student who developed pneumonia and was hospitalized. This suggests that the HAdV-B55 strain associated with this outbreak is not as virulent as those reported previously [25, 33], yet the strain should still be considered an urgent public health threat that necessitates measures to contain or control it in order to prevent epidemics.

Unlike other outbreaks caused by HAdVs in the northern regions of China [25, 27, 38] that occurred in the winter or spring, this outbreak in Beijing took place at the end of summer. Studies by Yu J., et al. and Liu T., et al. have reported that, although infections of HAdVs occur throughout the year, HAdV outbreak prevalence often peaks in winter and spring in the north of China and in summer and spring in the south of China [40, 41]. It is possible that HAdV outbreaks differ in their seasonality based on relative humidity and temperature. The fact that the HAdV outbreak reported here occurred in summer is thus significant because it could indicate this HAdV-B55 virus strain is capable of circulating in the climate and environmental reservoir.

The hexon, fiber, and penton genes of virus strain BJDX-01-2015 isolated in this study shared 100% sequence identity with those of the HAdV-B55 strain HAdV-B/CHN/BJ01/2011/55 (GenBank accession number JX491639), which was isolated from a patient with severe CAP in a previous study by Bin Cao et al. at Beijing in 2011 [42, 43]. The genome of BJDX-01-2015 is most similar to that of HAdV-B/CHN/BJ01/2011/55.

In our study, the strain of BJDX-01-2015 was obtained from the index case (ID#1), who presented with an upper respiratory infection. Furthermore most of the infected students were treated in outpatient clinics, and only one patient with non-severe pneumonia was hospitalized. The isolate of HAdV-B/CHN/BJ01/2011/55 was obtained from a patient with severe CAP in a previous study by Bin Cao et al. at Beijing [42], in which a total of 18 cases with laboratory-confirmed adenovirus infections, including the mentioned case with HAdV-B/CHN/BJ01/2011/55, were detected in 487 CAP cases. Furthermore, HAdV-B55 was most frequently detected in the 18 cases of adenovirus pneumonia (10/18), and six of the 10 patients ultimately developed acute respiratory distress syndrome. Based on the information of the two cases, two genetic viral samples, and the two independent studies that reported laboratory-confirmed ARIS with HAdV-B55 in Beijing, the isolate of BJDX-01-2015 led to milder ARIs.

These findings imply that severe ARIs were not simply caused by HAdV-B55 virus strains themselves but also depend on the conditions of the hosts, such as the individual’s age, general state of health, and the presence of co-morbidities or additional infections.

In this outbreak, we also found that for both the index case and the hospitalized case neutrophils increased and /or lymphocytes in the peripheral blood decreased. Studies on other respiratory viruses, such as influenza virus, respiratory syncytial virus, or human rhinovirus, have shown that an increase in neutrophils has a significant role in limiting virus replication [44, 45]. Thus, the increase in neutrophils observed here may have limited and resulted in milder clinical symptoms in this outbreak.

Most of the mutations at the amino acid level in BJDX-01-2015 were observed in the coding regions of protein VI. During the replication of HAdVs, protein VI functions as an adaptor to shuttle the hexon protein to the nucleus, where virus assembly occurs [46]. Whether these variations affect the virulence of BJDX-01-2015 and are responsible for the milder clinical symptoms observed in this study requires further investigation. We also found that BJDX-01-2015 has different numbers of poly “T” and poly “A” tracts compared with other HAdV-B55, HAdV-B11, and HAdV-B14 strains. The role that poly “T” and poly “A” tracts play in the evolution of HAdVs remains unclear and requires further research.

The increasing frequency of ARI outbreaks due to HAdV-B55 suggests that this re-emergent virus poses a serious threat to public health. It is therefore urgent that the local CDC improve epidemiological and virological surveillance of HAdV-B55.

## Conclusions

We identified HAdV-B55 as the cause of a recent localized ARI outbreak. This incident was the first reported outbreak in Beijing that can be attributed to this re-emergent virus. Our findings show that the risk of an HAdV-B55 epidemic should be paid more attention in Beijing, the capital of China, with a population of more than 20 million. Continuous surveillance of respiratory adenoviruses is an urgent need to understand the epidemiological and evolutionary features of HAdV-B55 and could also find value in an epidemiological modeling approach. We also found that the clinical laboratory data from this outbreak provides important reference for clinical diagnosis and may ultimately aid in informing the development of strategies to control and prevent respiratory tract infections caused by HAdV-B55 .

## Data Availability

The datasets used and/or analyzed in the current study are available from the corresponding author upon reasonable request.

## Abbreviations

PCR: Polymerase chain reaction
ARI: Acute respiratory infection
HAdV: Human adenovirus
ITR: Inverted terminal repeat
CDC: Center for disease prevention and control
CAP: Community-acquired pneumonia
UTR: Untranslated region
